# Decisional conflict and its determinants among patients with cancer undergoing immunotherapy combined with chemotherapy or targeted therapy: a cross-sectional study

**DOI:** 10.1038/s41598-023-39280-6

**Published:** 2023-08-05

**Authors:** Yun-Hsiang Lee, Xiao-Yin Chou, Yeur-Hur Lai, Yi-Hsin Liang, Chia-Tai Hung, Chu-Chi Hsaio, Zi-Xuan Gao

**Affiliations:** 1 School of Nursing, College of Medicine, National Taiwan University, Taipei, Taiwan; 2 Department of Nursing, National Taiwan University Hospital, Taipei, Taiwan; 3 Department of Nursing, Deh Yu College of Nursing and Health, Keelung City, Taiwan; 4 Department of Nursing, National Taiwan University Cancer Center, Taipei, Taiwan; 5 Department of Oncology, National Taiwan University Hospital, Taipei, Taiwan; 6 Department of Nursing, Mackay Medical College, New Taipei City, Taiwan

**Keywords:** Cancer, Oncology, Signs and symptoms

## Abstract

Decisional conflict might occur during shared decision-making (SDM) because immunotherapy is a rather novel treatment option for patients with cancer. To explore the prevalence and severity of physical and psychological symptoms and the effort invested in SDM in relation to decisional conflict among patients with cancer undergoing immunotherapy combined with chemotherapy or targeted therapy. This was a cross-sectional survey study. The SURE version of the Decisional Conflict Scale was used to screen cancer patients’ decisional conflict status. Demographic or clinical characteristics, physical symptoms and psychological distress; efforts invested in the SDM process were also assessed as potential factors related to decisional conflict. One hundred seventeen patients surveyed, the prevalence of fatigue (79.5%), sleep disturbance (78.6%), poor appetite (67.5%), and pain (58.1%) symptoms were high and the severity was at moderate levels. The prevalence of pruritus (40.2%), rash (34.2%), dry skin (41.9%), and diarrhea (17.1%) symptoms were low and the severity was at mild levels. 65.8% of patients reported uncertainty, with mild to moderate levels. Furthermore, 97.4% of the patients made some effort in SDM, and the effort level was moderate (mean: 5.56 ± 2.02). 64.1% of patients were certain that immunotherapy was the best option. Age, uncertainty, and effort in the SDM process were major factors related to decisional conflict. We observed that older patients (age: ≥ 65) and those with higher uncertainty levels and less effort in SDM reported higher levels of decisional conflict. Future studies should explore older patients’ decisional related needs of immunotherapy. Interventions should be designed to reduce the uncertainty experienced by patients with cancer and enhance their understanding of immunotherapy to enable them to take more effort in the SDM process.

## Introduction

Cancer is among the most life-threatening diseases worldwide^1^. With advances in medical technology, immunotherapy, which is a newly developed advanced treatment, is currently a crucial cancer treatment option^2,3^. Although immunotherapy provides hope^4^ for patients with cancer and an opportunity for them to prolong overall survival^5,6^, immunotherapy is not usually curative^7^. This causes the patients to be uncertain about how their disease would respond to immunotherapy, thereby leading to decisional conflict. Decisional conflict is defined in this study as uncertainty during clinical decision-making, which may lead to regret^8^. In cancer treatment, immunotherapy is often combined with chemotherapy or targeted therapy to enhance the clinical efficacy^9–11^. This may increase or complicate therapy-related adverse effects^12,13^, which can influence the patient’s decision to undergo immunotherapy. However, few studies have examined the relationships between therapy-related adverse effects and decisional conflict.

According to the Theory of Unpleasant Symptoms, patients with cancer generally have therapy-related adverse effects, including physical and psychological symptoms^14^, which may influence their decisional conflict. Physical symptoms, such as pain, fatigue, and insomnia, were reported to negatively influence decisional conflict in older patients with cancer undergoing treatment^15^. Furthermore, psychological distress, such as anxiety and depression, in patients with breast cancer can help predict their decisional conflict after an uninformative BRCA1/2 test^16^. For patients with cancer receiving therapy, the most common physical symptoms have been identified to include pain, fatigue, insomnia^17^, and poor appetite, along with those from immune-related adverse events (irAEs), such as pruritus (12.13–23.94%), diarrhea (11.16–27.95%), and rash (11.06–22.43%)^13^. A qualitative study including 28 patients with metastatic melanoma undergoing immunotherapy reported that uncertainty was the main form of psychological distress due to concerns about immunotherapy’s effectiveness and disease progression^18^. Uncertainty is an inherent characteristic of decisional conflict^8^. It can increase the patient’s cognitive load and reduce their ability to deploy the cognitive resources that they have, thereby impairing their cognitive appraisal abilities^19^. For example, patients may have difficulty determining the meaning of disease-related events when faced with uncertainty^20^. Thus, the patients might begin to doubt whether their original treatment decision is right, leading to decisional conflict^21^. Therefore, uncertainty was examined as a factors related to decisional conflict in the present study.

In clinical settings, to avoid decisional conflict, shared decision making (SDM) should be employed. In SDM, both clinicians and patients share their expertise when faced with the task of making decisions, and patients are supported and guided toward considering the range of options available to them to make an informed decision^22^. SDM is particularly important in clinical settings for achieving patient-centered care communication and thereby facilitating the delivery of high quality cancer care^23^. In fact, patients prefer to play a collaborative role in decision making^24^. However, previous studies have shown that only 35 ~ 50% of patients reported to have actually participated in the process of treatment through SDM^25–27^, and decisional conflict might have occurred. Thus, prior to decision making for anti-cancer treatment, patients should be informed of its benefits, risks, limitations, and uncertainties, and their preferences should be considered before making a final decision^28^, thereby reducing patients’ decisional conflict. SDM has been documented as a major influential factor related to conflict^29^. Therefore, it is crucial to assess the extent to which efforts have been invested in the SDM process for treatment through immunotherapy, which has not been sufficiently explored in prior research; the current study seeks to address this gap.

To our knowledge, this is the first study to simultaneously examine both major physical and psychological factors related to decisional conflict in patients with cancer undergoing immunotherapy. Hence, we hypothesized that patients with cancer undergoing immunotherapy may suffer from physical and psychological distress, which may influence the levels of decisional conflict, and that efforts invested in SDM could also influence the levels of decisional conflict. We determined the prevalence and severity of physical and psychological symptoms and effort invested in the SDM process in relation to decisional conflict among patients with cancer undergoing immunotherapy combined with chemotherapy or targeted therapy.

The results obtained from this study provide an important reference for health professionals to design specific interventions to decrease immunotherapy-related decisional conflict.

## Methods

### Study sample

The current study was cross-sectional, descriptive, and correlational in design. Data collection was conducted between February and November 2021. We recruited participants who were diagnosed with cancer and undergoing immunotherapy in inpatient oncology wards and chemotherapy outpatient settings of a medical center in northern Taiwan. Our assistant researcher screened for potential participants from electronic medical records of cancer patients who were receiving immunotherapy at inpatient oncology wards and chemotherapy outpatient settings in a medical center that we collaborated with. The inclusion criteria were as follows: being older than 19 years; having a diagnosis of cancer (stage III–IV); having received information from the physician about immunotherapy, such as it being a relatively new anticancer therapy and its adverse effects, risk, benefits, and financial cost; having received at least one dose of immunotherapy (because the median time of onset of certain irAEs, such as rash and pruritus, is 3 weeks after the first dose of immunotherapy)^30^; and being able to communicate in Mandarin or Taiwanese dialect. The exclusion criteria for participants were having cognitive functional impairment and the inability to respond to questions. Of the 131 potentially eligible patients, 5 were too tired, 2 had severe adverse effects, and 7 refused to participate because of concerns regarding the COVID-19 pandemic. Eventually, data from 117 patients were included in the final analysis (response rate = 89.3%).

### Data collection

#### The SURE version of the decisional conflict scale (SURE)

Patients’ level of decisional conflict was detected by the SURE version of the Decisional Conflict Scale, which has four items and has been designed for quickly identifying patients with clinically significant decisional conflict^31^. SURE is the acronym derived from four dimensions measured in this 4-item questionnaire (e.g., Sure of myself, Understand information, Risk–benefit ratio, and Encouragement). If the answer to an item is yes, the score is 1; if the answer is no, the score is 0. Thus, the four responses are added together to obtain a score ranging from 0 to 4, with a lower score indicating a higher degree of clinically significant decision making conflict; a total score of 4 indicates no decisional conflict. The SURE scale has acceptable internal consistency (Kuder-Richardson-20, coefficient = 0.7)^31,32^; its internal consistency (Cronbach’s alpha) in this study was 0.94.

#### Physical symptoms and psychological distress

Patients’ severity of physical symptoms and psychological distress were assessed using the 11-point numerical rating scale (NRS), ranging from 0 (“no pain”) to 10 (“the worst pain you can imagine”)^33^. The NRS is an acceptable response scale for assessing patient-reported outcomes^34^. For physical symptoms, we measured pain, fatigue, sleep disturbance, and poor appetite as the most common cancer-related symptoms^17^ and pruritus, rash, dry skin and diarrhea as the most common immunotherapy-related symptoms^13^. For the psychological aspect, we assessed the level of patients’ uncertainty in the same way. The internal consistency (Cronbach’s alpha) of the physical symptoms aspect in this study was 0.74, while that of the psychological distress (uncertainty) aspect was not presented due to our consideration of only one uncertainty item.

#### CollaboRate

Efforts toward facilitating the SDM process for immunotherapy was assessed using CollaboRate, a tool developed to measure efforts invested in the SDM process, and one that has demonstrated good psychometric properties^35^. We used CollaboRate based on the patient reports to revise three items to make it more specific to immunotherapy-related decision-making issues. For example, “How much effort was made to help you understand your health issues?” was revised to “How much effort was made to help you understand immunotherapy?” This item was scored on a 10-point anchored scale ranging from 0 (“no effort was made”) to 9 (“every effort was made”), with higher scores representing greater effort taken by patients to understand what immunotherapy is in the SDM process^35^. The internal consistency (Cronbach’s alpha) of the CollaboRate tool in this study was 0.87.

#### Sociodemographic and clinical characteristics

Patients’ sociodemographic characteristics included gender, age categorized based on the definition of elderly or not (≥ 65 or < 65)^36^, education in number of years classified according to education at or below the college or university level^37^, and marital status. Patients’ clinical characteristics included time since diagnosis, cancer type, treatment modules, treatment expenses (self-funded or non-self-funded, such as participation in clinical trials or insurance payments) and performance status, which was assessed by the Eastern Cooperative Oncology Group (ECOG) Performance Status Scale, with a score ranging from 0 (fully active) to 5 (dead)^38^.

### Statistical analysis

Data analysis was performed using SPSS version 24.0 software. Descriptive statistics including percentage, mean, and standard deviation [SD] were used to present participants’ sociodemographic and clinical characteristics, and the current status of physical symptoms (pain, fatigue, poor appetite, and sleep disturbance), psychological distress (uncertainty), efforts invested in the SDM process and decisional conflict (aim 1). Since some scores of measures were not normally distributed, we employed nonparametric analysis methods to present the inference statistics. The levels of decisional conflict were dichotomized as non-conflict cases (the SURE total score was 4) versus cases having some level of conflict (the SURE total score was 0, 1, 2, or 3). Before conducting logistic regression, the Mann–Whitney U test was performed to compare the differences of potential factors (independent variables) in the levels of decisional conflict. Further, Spearman’s tests were conducted to determine the potential factors correlated with decisional conflict. All the significant potential factors were included in a logistic regression model^39^ to determine the robust factors related to decisional conflict (aim 2).

### Sample size

We strived to have sufficient samples by using G-Power with alpha = 0.05 and power = 0.8 in a two-sided test and adopted a giving odds ratio (OR) of 2.09 based on previous studies^40^ in conducting the logistic regression, to reasonably detect an effect in the current study^41^. The minimum sample size of 101 was suggested. Thus, 117 participants were considered adequate to conduct the logistic regression in this study.

### Ethics approval

The study was conducted in accordance with the Declaration of Helsinki to follow the principles of Ethical Considerations. This study was approved by the Institutional Review Board of National Taiwan University Hospital, Taipei, Taiwan (approval no. 201812191RINB). The participants were informed about the study purpose, after informed consent was obtained from all subjects; subsequently, paper-based questionnaires were distributed.

### Consent to participate

All participants provided written informed consent prior to enrolment in the study.

## Results

### Patients’ sociodemographic and clinical characteristics and their relationships with decisional conflict

Most of the patients were male (78.6%). The average age of patients was 56.1 (SD = 11.2) years and the average education years was 11.9 (SD = 3.9). Most patients were married (76.9%) and more than half of them (59.0%) were diagnosed with cancer, for more than or equal to 12 months. Furthermore, 51.3% of the patients were diagnosed with head and neck cancer while other cancer types were very diverse (48.7%), such as lung cancer, hepatocellular carcinoma (HCC), gastrointestinal stromal tumor (GIST), breast cancer, and colon cancer. Moreover, 76.9% of the cancer patients who were receiving immunotherapy reported receiving treatment combined with chemotherapy or targeted therapy. 61.5% of patients reported self-funding for immunotherapy and the mean performance status (ECOG) for patients was 1.43 (SD = 0.83) (Table 1).

**Table 1:** Patients’ sociodemographic and clinical characteristics and their relationships with decisional conflict (N = 117).

Characteristics	n (%)	Mean (SD)	Levels of decisional conflict		
			Mean (SD)	Mean rank	z
Gender					
Male	92 (78.6)		2.70 (1.62)	59.93	− 0.61
Female	25 (21.4)		2.60 (1.58)	55.58	
Age (years)		56.1 (11.2)			− 3.73**
< 65	87 (74.4)		3.97 (1.52)	65.37	
≥ 65	30 (25.6)		1.80 (1.54)	40.63	
Education (years)		11.9 (3.9)			
Below college	74 (63.2)		2.31 (1.67)	51.94	− 3.18**
College or university	43 (36.8)		3.30 (1.26)	71.15	
Marital status					
Married	90 (76.9)		2.60 (1.59)	65.13	− 1.15
Unmarried	27 (23.1)		2.93 (1.66)	57.16	
Time since diagnosis					
< 12 month	48 (41.0)		2.54 (1.62)	56.79	− 0.63
≧12 month	69 (59.0)		2.77 (1.59)	60.54	
Cancer type					
Head and neck cancer	60 (51.3)		2.83 (1.45)	60.93	− 0.68
Other (lung, HCC, GIST, breast, colon, pancreas, gall bladder)	57 (48.7)		2.51 (1.74)	56.96	
Treatment					
Only immunotherapy	27 (23.1)		2.70 (1.46)	57.06	0.73
Immunotherapy + chemotherapy	31 (26.5)		2.68 (1.70)	60.1	
Immunotherapy + targeted therapy	36 (30.7)		2.81 (1.60)	61.82	
Immunotherapy + chemotherapy + targeted therapy	23 (19.7)		2.43 (1.70)	55.39	
Dose of treatment					
1–3 doses	68 (58.1)		2.59 (1.66)	57.78	0.57
4–6 doses	22 (18.8)		3.00 (1.41)	65.34	
> 6 doses	27 (23.1)		2.63 (1.62)	56.91	
Treatment expense					
Self-funded	72 (61.5)		2.9 (1.5)	55.65	− 1.46
Non-self-funded	45 (38.5)		2.5 (1.6)	64.37	
			Correlations with decisional conflict		
					r
Performance status (by ECOG)		1.43 (0.83)			− 0.22*

Mann–Whitney U test was performed to compare the differences of potential factors (independent variables) in the levels of decisional conflict. Spearman’s correlation tests were conducted to determine potential factors related to decisional conflict.

*HCC* Hepatocellular carcinoma; *GIST* Gastrointestinal stromal tumor; *ECOG* Eastern cooperative oncology group.

*p < 0.05, **p < 0.01.

We found the differences in the levels of decisional conflict among patients’ age (≥ 65 vs. < 65) (Z = − 3.73, *p* < 0.01) and education (college or university level vs. below college) (Z = − 3.18, *p* < 0.01). This indicated that older patients (aged ≥ 65 years) and those having lower level of education reported higher levels of decisional conflict than younger patients (aged < 65 years) and those with college or university level of education. Patients’ performance status were negatively associated with decisional conflict (r = − 0.22, *p* < 0.05) (Table 1).

### Prevalence and severity of physical symptoms and psychological distress and efforts invested in the SDM process: correlations with decisional conflict

With respect to the prevalence of patients’ physical symptoms, the prevalence of immunotherapy-related symptoms such as for pruritus, rash, dry skin, and diarrhea were 40.2%, 34.2%, 41.9%, and 17.1%, respectively, and for cancer-related symptoms such as pain, fatigue, poor appetite, and sleep disturbance were 58.1%, 79.5%, 67.5%, and 78.6%, respectively. Furthermore, the mean scores for pruritus, rash, dry skin, diarrhea, pain, fatigue, poor appetite, and sleep disturbance were 1.95 (SD = 2.61), 1.03 (SD = 2.04), 1.09 (SD = 2.05), 1.17 (SD = 2.11), 3.12 (SD = 3.31), 4.06 (SD = 3.01), 3.27 (SD = 3.03), and 4.05 (SD = 3.10), respectively. The prevalence of patients’ uncertainty was 65.8%, and the mean uncertainty levels was mild (2.86; SD, 2.73). Moreover, 97.4% of the participants made some effort in SDM, with the mean effort levels being moderate (5.56; SD, 2.02) (Table 2).

**Table 2:** Prevalence and severity of physical symptoms and psychological distress and efforts invested in the SDM process: correlations with decisional conflict (N = 117).

Independent variables	Prevalence	Severity/levels	Correlations with decisional conflict
	N (%)	Mean (SD)	r
Physical symptoms (0–10)			
Immunotherapy-related symptoms			
Pruritus	47 (40.2)	1.95 (2.61)	− 0.08
Rash	40 (34.2)	1.03 (2.04)	− 0.01
Dry skin	49 (41.9)	1.09 (2.05)	− 0.1
Diarrhea	20 (17.1)	1.17 (2.11)	− 0.1
Cancer-related symptoms (0–10)			
Pain	68 (58.1)	3.12 (3.31)	− 0.21*
Fatigue	93 (79.5)	4.06 (3.01)	− 0.30**
Poor appetite	79 (67.5)	3.27 (3.03)	− 0.29**
Sleep disturbance	92 (78.6)	4.05 (3.10)	− 0.1
Psychological distress 0–10)			
Uncertainty	77 (65.8)	2.86 (2.73)	− 0.39**
Effort invested in the SDM process (0–9)	114 (97.4)	5.56 (2.02)	0.20*

Physical and psychological symptoms were measured using the NRS (Numerical Rating Scale); Effort invested in the SDM process was measured using the CollaboRate tool; Decisional conflict was measured using the SURE (SURE version of the Decisional Conflict Scale; *SDM* Shared decision making; Spearman’s correlation tests were conducted to determine potential factors related to decisional conflict.

*p < 0.05, ** *p* < 0.01.

Only cancer-related symptoms such as pain (r = − 0.21, *p* < 0.05), fatigue (r = − 0.30, *p* < 0.01), and poor appetite (r = − 0.29, *p* < 0.01) were negatively associated with decisional conflict. Patients’ uncertainty (r = − 0.39, *p* < 0.01) was negatively associated with decisional conflict as well. Moreover, patients’ efforts invested in the SDM process (r = 0.20, *p* < 0.05) was positively associated with decisional conflict (Table 2).

### Status of immunotherapy-related decisional conflict

For the status of decisional conflict, 64.1% of patients reported that they felt sure about immunotherapy being the best choice (i.e., response on the Sure of myself item); the response rates for the other 3 items of the SURE scale about Understand information, Risk–benefit ratio, and Encouragement were 71.8%, 70.9%, and 60.7%, respectively. Results revealed that 80.3% of patients experienced clinically significant decisional conflict (total scores less than 4) (Table 3).

**Table 3:** Status of immunotherapy-related decisional conflict (N = 117).

Four items of SURE		No	Yes
		n (%)	n (%)
Sure of myself	Do you feel SURE about the best choice for you?	42 (35.9)	75 (64.1)
Understand information	Do you know the benefits and risks of each option?	33 (28.2)	84 (71.8)
Risk–benefit ratio	Are you clear about which benefits and risks matter most to you?	34 (29.1)	83 (70.9)
Encouragement	Do you have enough support and advice to make a choice?	46 (39.3)	71 (60.7)
		Decisional conflict	
Total SURE^a^		No	Yes
		n (%)	n (%)
		23 (19.7)	94 (80.3)

^a^Patients who received total scores less than 4 experienced clinically significant decisional conflict.

### Factors related to decisional conflict

For the decisional conflict model, significant factors related to conflict included age, uncertainty, and the effort invested in the SDM process (Table 4). The OR for conflict risk was 4.82 times higher (95% CI 1.48–15.69) in older patients than in younger patients. Moreover, the OR for conflict risk was higher if the patients had higher levels of uncertainty (95% CI 1.04–1.52) and lower if they spent more effort investing in the SDM process (95% CI 0.58–0.96; Table 4).

**Table 4:** Factors Related to Decisional Conflict and Analyzed Using Multivariate Logistic Regression (N = 117).

Variable	Beta (SE)	Odds ratio	*p*	95% CI
Age (≥ 65 vs. < 65)	1.57 (0.60)	4.82	0.009	1.48–15.69
Education (below college vs. college or university)	− 0.49 (0.47)	0.61	0.300	0.24–1.55
ECOG	0.08 (0.31)	1.08	0.801	0.59–2.00
Pain	0.11 (0.08)	1.11	0.187	0.95–1.30
Fatigue	0.01 (0.09)	1.01	0.889	0.84–1.23
Poor appetite	0.07 (0.09)	1.07	0.430	0.90–1.27
Uncertainty	0.23 (0.10)	1.26	0.018	1.04–1.52
Effort invested in the SDM process	− 0.29 (0.13)	0.75	0.022	0.58–0.96

*ECOG* Eastern cooperative oncology group; the ECOG Scale was used to assess performance status; *SDM* Shared decision making.

## Discussion

In this cross-sectional study, 76.9% of the cancer patients who were receiving immunotherapy reported receiving treatment combined with chemotherapy or targeted anti-cancer agents, an approach supported by recent clinical trials for improved clinical efficacy^9–11^. Thus, patients with cancer might experience a variety of physical symptoms from a combination of different therapies. In the current study, approximately 80% of patients experienced fatigue (79.5%) and sleep disturbance (78.6%) symptoms, and over half of the patients had poor appetite (67.5%) and pain (58.1%) symptoms. In an earlier systematic review of patients who were undergoing surgery, chemotherapy, or radiotherapy, the prevalence of physical symptoms varied across different studies examining fatigue (38.9–82.1%), sleep disturbance (14–72.2%), poor appetite (2.0–76.2%), and pain (17.4–61.8%)^13^. One point of clarification: this review study did not include patients who had undergone immunotherapy. The review study included patients with all stages of cancer who had received surgery, chemotherapy or radiotherapy. One noteworthy finding was that patients with cancer stages III and IV, especially those undergoing chemotherapy, exhibited a higher prevalence of physical symptoms. The lower range data is attributed to those patients with cancer stages I and II. Moreover, both the systematic review and the current study indicate similarities in which patients with cancer stage III or IV have a higher prevalence of physical symptoms. However, in the current study, only patients with cancer stages III and IV were observed. In closing, clinicians should exercise caution when caring for these patients in particular. Presently, there is an inadequate amount of study exploring whether or not immunotherapy (as only treatment or in combination with other treatment options) leads to a higher prevalence of physical symptoms compared to those patients who do not receive immunotherapy. Thus, future studies are recommended to focus on these issues.

Moreover, over one-third of the patients reported pruritus (40.2%), rash (34.2%), and dry skin (41.9%) symptoms, and approximately one-fifth reported diarrhea (17.1%). These prevalence are higher than those reported by a past systematic and meta-analysis study^13^ examining the immune-related side effects of pruritus (12.13–23.94%), rash (11.06–22.43%), dry skin (5.42%), and diarrhea (11.16–27.95%)^13^. The difference may be because, in the current study, most (76.9%) of the patients were undergoing immunotherapy combined with targeted therapy and those symptoms were also more common in patients with targeted therapy, with over 50% of patients experiencing skin toxicities (pruritus, rash, and dry skin)^42^ and diarrhea^43^. Some similarities in side effects can be observed between immunotherapy and targeted therapy. Although we found that treatment modalities were not associated with decisional conflict in this study, health-care professionals should pay more attention to such a high prevalence of symptoms in patients with cancer and provide appropriate and adequate treatment. The management of targeted therapy and immunotherapy does not involve the same measures. Using sunscreen with a sun protection factor (SPF) of at least 15 to protect the skin from the sun’s harmful rays, a mild soap for managing skin problems^44^, and drinking enough water to prevent dehydration^45^ are common recommendations for patients. Furthermore, diarrhea may also result from chemotherapy. However, according to the management of immunotherapy guidelines, corticosteroids may routinely be used to manage irAEs^46^. Thus, cancer patients demonstrating any symptoms would benefit from engaging in discussions with the health care team to distinguish possible causes of the problem. Furthermore, regarding symptom severity, health providers should pay more attention when patients report more than moderate levels of symptoms, since most of the patients experience intolerable moderate and severe symptoms^47^, especially related to fatigue and sleep disturbance.

We also found that approximately 70% of patients knew the benefits and risks of immunotherapy, indicating that they were aware of their treatment choice as it was described to them. Even though 64.1% of patients reported feeling that immunotherapy was the best choice, the implementation of SDM still requires more efforts. Approximately 60% of the patients in this study reported having enough support and advice to make an informed choice, consistent with several studies reporting that 35–50% of patients were involved in treatment-related SDM^25,26^. However, this SURE item merely indicates the patient’s subjective perception of having received sufficient support and advice in SDM. More research is required on specific items that are insufficient.

Furthermore, three factors associated with immunotherapy-related decisional conflict were found: age, uncertainty, and effort in the SDM process. We found that patients  ≥ 65 years showed a high risk of decisional conflict. Most older patients may experience mild age-associated changes in cognition, which is a normal part of aging process but affects thinking speed and attention^48^. This may cause them to take longer to respond to or understand immunotherapy to avoid or reduce decisional conflict. Further, elder people tend to seek less information when making decisions^49^, especially if they are both physically and mentally affected by cancer. Although health-care providers should actively provide information on immunotherapy to older patients and give them sufficient time to understand the information provided, the context of the SDM must be considered^28^. The patients should first be asked about the depth of information that they seek. If they deliberately choose not to be informed or to be minimally informed, health-care providers must consider the patients’ preferences while still ensuring that patients make informed decisions.

Our finding on higher levels of uncertainty presenting a higher risk of conflict was consistent with a prior report which documented that unaddressed uncertainty can result in treatment-related decisional conflict^21^. Moreover, a study indicated that since most medical decisions are complicated by a lack of evidence about risk/benefit information, decision satisfaction is related to the uncertainty^50^. Thus, in patients facing new immunotherapy, uncertainty may persist in their minds regardless of increased medical knowledge^51^. Indeed, our study provided quantitative results of the high prevalence (65.8%) of uncertainty, which was reported by cancer patients undergoing immunotherapy and was supported by a previous qualitative study^18^. Although the severity level of uncertainty was mild in this study, it is one of the major factors in decisional conflict. Therefore, understanding the reasons for uncertainty and assessing the needs of patients are recommended steps in the SDM process to help them reduce or cope better with their feelings of uncertainty.

Furthermore, in our study, patients reported that they invested moderate levels of effort trying to understand immunotherapy. In addition, while they invested more effort in understanding immunotherapy, a higher efforts invested in the SDM process among patients resulted in lower risk of decisional conflict in this study. This result is consistent with a previous study^29^ which showed that patients who perceived that they were more involved in the SDM process were significantly negatively correlated with decisional conflict. Thus, since a high prevalence (80.3%) of immunotherapy-related decisional conflict was found in this study, it is strongly recommended that health professionals should design specific interventions to decrease patients’ uncertainty and encourage them to involve in the discussion of immunotherapy for reducing decisional conflict, which would reflect better communication in patient-centered care and the performance of SDM^23^.

Lastly, some interesting findings from this study were that several factors in the correlation analysis, including severe physical performance, severe pain, fatigue, and poor appetite, that were associated with greater immunotherapy-related decisional conflict. These factors were newly explored in this study, especially for physical symptoms related to decisional conflict, supported by our hypothesis. However, these factors were not related to immunotherapy-related decisional conflict. Uncertainty as a factor of psychological distress in this study was related to decisional conflict. Some factors can explain these results. Most cancer patients experience physical symptoms (such as pain, fatigue, and poor appetite) for a long time during their anti-cancer therapies^12,13^ that could be managed in clinical settings^52^, so that they could tolerate these symptoms. In addition, immunotherapy is commonly used as a second-line or later treatment modality, and thus it can provide renewed hope for patients^4^. At that point, they would focus on the efficacy of treatment and how long they will survive, and these issues are uncertain^7^. Thus, for avoiding decisional conflict, uncertainty has to be managed in ways such as collaborating with doctors to provide the knowledge to increase immunotherapy-related information and invite patients to participate more in the SDM process, which reflects another important factor related to decisional conflict in this study.

## Limitations

This study has some limitations. First, only 23.1% of the patients received immunotherapy in this study, and most of them received immunotherapy combined with chemotherapy or targeted therapy. Thus, a further analysis of decisional conflict-related factors in different immunotherapy modalities could be considered using larger sample sizes. Second, men were overrepresented in our cohort and most patients had head or neck cancer, thus precluding the generalization of these findings to patients with other cancers, such as breast cancer. Third, we collected data on the most common immunotherapy-related adverse effects, namely, pruritus, rash, dry skin, and diarrhea; however, we did not have an “others” option. Thus, we could not evaluate other immunotherapy-related adverse effects, such as cardiovascular, hematologic, renal, and neurological symptoms^53,54^. Four, we could assess only the level of decisional conflict in the current study. A qualitative study is warranted on how the patient’s immunotherapy experience affects decisional conflict. Finally, self-reported CollaboRATE may have social desirability bias^55^ and a priori dichotomization of age needed to consider the estimated risk factor effect was biased^56^ in the current study.

## Conclusion

Immunotherapy is a newly developed anti-cancer therapy. The present study examined the factors associated with immunotherapy-related decisional conflict. Results revealed that age, uncertainty, and effort invested in the SDM process were major factors related to decisional conflict. It is strongly recommended to design interventions that can support cancer patients to reduce or cope better with uncertainty, and invite patients and encourage them to spend more time and efforts in the SDM process. Further, proactive care should be taken for cancer patients ≥ 65 years, who may experience more decisional conflict and need more assistance from health professionals for reducing the same.

## Data Availability

The datasets used and/or analyzed during the current study available from the corresponding author on reasonable request.
